# Supplementary material for: Characterization of Gallibacterium anatis Isolated from Pathological Processes in Domestic Mammals and Birds in the Czech Republic
Source: Pathogens. 2024 Mar 7;13(3):237. doi: 10.3390/pathogens13030237 (PMC10975909; doi:10.3390/pathogens13030237)
Supplement: Supplementary file 1 [file pathogens-13-00237-s001.zip › Supplement1_MLST.pdf]

Table S1. Table of *Gallibacterium anatis* new sequence types

Isolate number	Country	Year of isolation	Symptom	Source	Animal	Genes								
						adk	*atpD*	*fumC*	*gyrB*	*infB*	*mdh*	*recN*	*thdF*	*ST*
H10	Czechia	1975	Multi-organ infection	Reference strain	Hen	8	32	51	52	41	33	42	34	74
H7	Czechia	2017	Multi-organ infection	Spleen	Hen	29	28	52	53	43	35	44	35	83
H8	Czechia	2017	Multi-organ infection	Spleen	Hen	18	6	48	54	44	36	45	8	84
H9	Czechia	2017	Multi-organ infection	Spleen	Hen	16	6	48	54	44	36	45	8	85
G10	Czechia	2018	Multi-organ infection	Cloaca	Hen	27	29	47	49	20	30	38	31	86
G11	Czechia	2018	Multi-organ infection	Trachea	Hen	27	29	47	49	20	30	38	31	86
G12	Czechia	2018	Diarrhoea	Faeces	Calf	10	30	48	50	38	31	39	32	88
G6	Czechia	2019	Diarrhoea	Intestine	Calf	10	30	49	50	38	31	39	32	89
G7	Czechia	2019	Diarrhoea	Rectum	Calf	10	30	48	50	38	31	39	32	88
G8	Czechia	2019	Diarrhoea	Intestine	Calf	10	30	50	51	39	31	40	33	91
G9	Czechia	2019	Diarrhoea	Intestine	Calf	28	31	48	17	40	32	41	8	92
H11	Czechia	2019	Diarrhoea	Intestine	Calf	10	30	49	51	42	34	43	33	93
H12	Czechia	2019	Diarrhoea	Intestine	Calf	10	30	49	50	38	31	39	32	89
